# Hydraulic Properties of a Rock‐Soil‐Root System: Insights From *Fraxinus ornus* L. Saplings Growing on Different Carbonate Rocks

**DOI:** 10.1111/pce.15369

**Published:** 2025-01-08

**Authors:** Sara Di Bert, Martina Tomasella, Patrick Duddek, Sara Natale, Francesco Petruzzellis, Andrea Carminati, Luca Zini, Lorenzo D'Amico, Giuliana Tromba, Andrea Nardini

**Affiliations:** ^1^ Physics of Soils and Terrestrial Ecosystems, Department of Environmental Systems Science, Institute of Terrestrial Ecosystems ETH Zürich Zurich Switzerland; ^2^ Dipartimento di Scienze della Vita Università di Trieste Trieste Italia; ^3^ Dipartimento di Biologia Università di Padova Padova Italia; ^4^ Dipartimento di Matematica, Informatica e Geoscienze Università di Trieste Trieste Italia; ^5^ Elettra‐Sincrotrone Trieste, Area Science Park, Basovizza Trieste Italia; ^6^ School of Physics and Astronomy Monash University Clayton Victoria Australia

**Keywords:** bedrock, drought, plant hydraulics, root

## Abstract

Drought impacts trees in varied temporal and spatial patterns, suggesting that heterogeneity of below‐ground water stores influences the fate of trees under water stress. Karst ecosystems rely on shallow soil overlying bedrock that can store available water in primary pores. A contribution of rock moisture to tree water status has been previously demonstrated, but actual mechanisms and rates of rock‐to‐root water delivery remain unknown. We report accurate measurements of hydraulic properties of two rock types (Breccia and Dolostone), of typical Karst red soil, and of roots of a common Karst tree species grown under different rock‐soil combinations. Experimental data were used to build a water exchange model that supported the hypothesis that roots can extract water from porous and highly conductive rocks (Breccia), but not from more compact ones (Dolostone), especially when plants grow in rocky substrate or experience water stress, and thus have low root hydraulic conductivity and low rates of water extraction from rocks. Our data support the hypothesis that rocks represent important water stores for plants growing in rock‐dominated habitats. Heterogeneous rock properties translate into different rates of water delivery to root systems, underlying complex patterns of tree mortality under severe drought stress.

## Summary statement

- Plant roots can extract water from porous and highly conductive rocks (Breccia), but not from more compact ones (Dolostone), especially when plants grow in rocky substrate or experience water stress, and thus have low root hydraulic conductivity and low rates of water extraction from rocks.
- Our data support the hypothesis that rocks represent important water stores for plants growing in rock‐dominated habitats, and that heterogeneous rock properties translate into different rates of water delivery to root systems.

## 1 Introduction

Ongoing climate change is increasing the frequency and severity of drought events, posing challenges to plant survival whenever the balance between water loss to the atmosphere and water uptake from belowground stores is severely compromised (McDowell et al. 2022). Global‐change driven droughts have already produced profound impacts on vegetation (Allen et al. 2010, Choat et al. 2012), leading to widespread tree mortality across multiple forest ecosystems (Hartmann et al. 2022). These mortality events are often intensified by heatwaves and can be further compounded by fires, pests, pathogens, and other environmental stressors (McDowell et al. 2022). The precise mechanisms behind drought‐induced tree mortality remain a topic of debate, but there is a general consensus linking drought to water stress, hydraulic failure, and subsequent plant death (Nardini, Battistuzzo, and Savi 2013, Adams et al. 2017, Choat et al. 2018). Other factors, like carbon starvation (McDowell et al. 2011), are likely to play additional roles, in particular during the eventual post‐drought recovery phase (Klein et al. 2018, Nardini et al. 2018, Tomasella et al. 2020).

Drought‐induced tree mortality occurs in varied spatial and temporal patterns, which might be attributed to factors like inter‐ and intraspecific morphological and physiological traits, competition, pests, or edaphic and climatic drivers (MacGregor and O'Connor 2002, De Toledo et al. 2011, Baguskas et al. 2014, Petrucco et al. 2017, Savi et al. 2019). Deep‐rooted species can survive drought by accessing deep water that is better protected from evaporation to the atmosphere and less exploited by root systems of neighbouring shallow‐rooted species (Nardini et al. 2016, Johnson et al. 2018), although this advantage can be overcome by multi‐annual droughts that prevent recharge of deep water stores (Chitra‐Tarak et al. 2018). However, large portions of terrestrial habitats rely on soils less than 50 cm thick, overlying bedrock that limits root growth and water storage (Shangguan et al. 2017). Nevertheless, when such shallow soils overlie fractured bedrock, roots can penetrate and eventually tap into rock moisture (Zwieniecki and Newton 1995, Schwinning 2010, Nardini, Tomasella, and Di Bert 2024), and these observations call for a better understanding of heterogeneity of belowground water storages and their availability to plants over prolonged and intense drought.

Various non‐soil substrates, such as weathered granite, epikarst and petrocalcic horizons, store significant water volumes (Graham et al. 1997, Querejeta et al. 2006). Different studies have revealed that even compact bedrock contains a significant percentage of water potentially available to plants. This water is held in the primary pores of the rock at water potential values above the turgor loss point of roots (Schoeman, Kruger, and Loock 1997, Korboulewsky et al. 2020, Nardini et al. 2021). Due to the relative volumes of soil and bedrock, in some habitats rock moisture represents the major water reservoir accessible to root systems. As an example, Nardini et al. (2021) investigated a 5 m deep system in a limestone‐dominated Karst area. The top 70 cm of the belowground profile were occupied by soil, overlying compact bedrock. Based on relative volumes and available water contents of soil and rocks, it was calculated that the system could store a total of 190 mm of water potentially available to plants, of which only 60 mm were located in the soil and the remaining 130 mm were stored in the bedrock's primary pores. Similarly, Rempe and Dietrich (2018) calculated that nearly 30% of annual precipitation is stored in the bedrock, sustaining Mediterranean vegetation in a site in northern California.

There is also evidence suggesting that plants use this resource to survive drought in seasonally dry habitats. In the southern Sierra Nevada, co‐occurring Jeffrey pine (*Pinus jeffreyi*) and Greenleaf manzanita (*Arctostaphylos patula*) tapped into deep water sources, including rock moisture, as seasonal drought progressed (Rose, Graham, and Parker 2003). Hahm et al. (2020) showed that oaks growing in northern California depleted rock moisture during summer drought, and similar results were obtained by Montaldo et al. (2021) investigating olive trees and oaks growing in a Mediterranean site with shallow soil overlying basalt bedrock. Nardini et al. (2021) also found that trees growing over porous limestone rocks retained a better water status during summer drought compared to those growing over compact dolostone.

While there is evidence that rock moisture is an important water source for plants in several forest ecosystems (McCormick et al. 2021), it is still largely unknown how and to what extent plants can efficiently extract water from rock reservoirs. Roots often occupy rock fractures with water potentially stored in the adjacent matrix (Nardini, Tomasella, and Di Bert 2024) or develop layers over the soil‐bedrock interface (Hellmers et al. 1955). However, research on actual mechanisms of hydraulic interaction between rocks and roots remains limited. Schwinning (2020) discussed the possible pathways for water transfer from rocks to roots, including direct contact and water delivery from rock surface to roots, soil‐mediated water transfer, or direct exploration of rock pores by mycorrhizal hyphae (see also Nardini, Tomasella, and Di Bert 2024). While all these hypotheses are reasonable from a qualitative point of view, it should be stressed that water transfer from rocks to roots, either direct or mediated by soil or fungal hyphae, is only possible and functionally relevant when the hydraulic conductivities of these different compartments are of similar order of magnitude to allow water flowing at physiologically useful rates. Hence, comparing the hydraulic properties of different rock types with those of surrounding soil and roots of species growing over a specific soil‐rock system appears as a crucial step toward a better quantitative understanding of rock‐root water relations. In this context, plant physiologists face the problem that rock hydraulic properties are generally measured at spatial scales and with experimental methods that are not fully relevant for the study of plant water relations at the cell, tissue or organ levels. Hence, the present study was designed to provide a detailed characterisation of hydraulic properties of roots, soil and rocks in an experimental system mimicking the possible rock‐soil‐root interactions in the substrates found in the Classical Karst region (Nardini et al. 2021). We specifically aimed at testing different hypotheses based on a set of experimental measurements. Using microCT imaging techniques, hydraulic measurements and estimates of water potential changes as a function of water content, we tested whether limestone rocks with high porosity have higher connectivity of water pathways and thus higher hydraulic conductivity compared to compact dolostones. We further aimed at comparing hydraulic properties of rocks with those of red soil typical of the Classical Karst region. Secondly, we hypothesised that root hydraulic conductivity of saplings of a prevalent Karst tree species is generally higher than rock hydraulic conductivity, but it declines under drought conditions thus approaching values closer to the ones measured for rocks. To this aim, plants were grown in rock‐free soil or in mixtures of soil and rock fragments, either under well‐watered conditions and during an experimental drought. We finally tested the hypothesis that rocks can actually deliver water to the roots, but at different rates according to rock type, plant and soil water status, and eventual direct root‐rock contact. To test this, we developed a computational model to estimate the efficiency of water exchange across various spatial rock‐soil‐root arrangements, under simulated progression of drought conditions. Our final aim was to test under which conditions rock‐to‐root water delivery is thermodynamically possible and quantitatively meaningful.

## 2 Materials and Methods

One‐year old saplings of *Fraxinus ornus* L., sourced from a public nursery (Vivai Pascul, Tarcento, Italy), were used in experiments involving two Cretaceous carbonate lithotypes, that is, Breccia (B) and Dolostone (D) from the Monrupino formation (Jurkovšek et al. 2016), and local red soil. Plants were grown in the nursery from seeds in 0.25 L pots and maintained in the open under a shading net intercepting 30% of solar radiation. Plants were irrigated daily to pot capacity. Rock samples were collected in February 2021 from two nearby sites near Sgonico, Italy (coordinates: 45° 44′ 12.1′′ N ‐13° 44′ 56.4′′ E), in a deciduous woodland in the Classical Karst Plateau (Nardini et al. 2021). Rocks were sampled from two different sites dominated by B or D bedrock, and large rock fragments (about 5–10 L in volume) were transported to the laboratory for preparation of sub‐samples (see below). The red soil was sourced from a nearby vineyard (Savi et al. 2018).

Saplings were transplanted into 0.75 L pots after carefully cleaning the roots from the potting mix used in the nursery. Plants were divided in three groups with different mixtures of soil and rock fragments (13 plants per group), in different volume percentages: (i) 60% red soil mixed with 40% Breccia fragments (group B); (ii) 60% red soil mixed with 40% Dolostone fragments (group D); (iii) 100% red soil (group S). Breccia and Dolostone rock samples were fractured to obtain samples with a volume of 10–60 cm^3^. Rock fragments were sunk in water for 24 h before potting to favour water saturation of the porous spaces. All plants were fertilised after transplanting with 1 g of slow‐release fertiliser (Flortis Multiporpuse EC Fertiliser, Ortivital SpA, Milano, Italy). Plants were grown in a greenhouse, irrigated from the top twice a day at pot capacity and randomly shifted weekly to assure uniform radiation exposure among groups. The initial sapling height and base diameter were recorded at transplanting. Experiments started 3 months after transplanting.

### 2.1 Hydraulic Properties of Soil and Rocks

We used the HYPROP evaporation technique (Peters and Durner 2008) and the WP4C dew point hygrometer (METER Group Inc. Pullman, WA, USA) to measure soil water retention and unsaturated hydraulic conductivity (k_soil_) (Diouf et al. 2020). Three soil samples with a bulk density of 1.08 g cm^−^^3^ were measured with the HYPROP system. Each main sample yielded three sub‐samples for dew point measurements, for a total of nine replicates. During controlled dehydration, we measured changes in soil water potential and fresh weight with the WP4C and a SPU123 analytical balance (Ohaus Scout ProBalance, Parsippany, NJ, USA). After that, samples were oven‐dried at 105°C for 24 h. We assessed saturated hydraulic conductivity on three similar samples using the KSAT (METER Group Inc. Pullman, WA, USA) and a falling head approach (De Jong van Lier, de Melo, and Pinheiro 2024). The data were analyzed with the HYPROP‐FIT software (UMS GmbH Munich) based on the bi‐modal Mualem‐van Genuchten model (Supporting Information S1: Figure S1) (Durner et al. 1999).

To quantify the efficiency of water flow through the rock primary pores, we measured saturated rock hydraulic conductivity (*k*_rock_, kg m^−^^1^ s^−^^1^ MPa^−^^1^) of both D and B samples. Five cylindrical rock samples per rock type, 4 cm long and 2.5 cm in diameter, were carved from square blocks. Rock samples were immersed in distilled water for 24 h while subjected to a −60 kPa vacuum pressure to fully rehydrate their pores. Accurate measurements of length and transverse surface area were taken before the samples were inserted into compression couplings for High Pressure Flow Meter (HPFM, Dynamax Inc., Houston, TX, USA) attached to a chromatography capillary, and immersed in a beaker filled with distilled water. The setup was then placed in a pressure chamber (Supporting Information S1: Figure S2), with the capillary protruding outside. Air pressure (0.3 MPa for Dolostone and 0.2 MPa for Breccia) was applied in the pressure chamber to force water flow through the rock samples while optimising flow rates and water collection time based on preliminary experiments. Water mass flowing out of the capillary was collected in pre‐weighed Eppendorf tubes filled with sponge at 10 min intervals, with each measurement lasting 5 min. Each session lasted 90 min, with stable flow reached after 30 min, so that only values recorded after 30 min were used in the calculation of maximum hydraulic conductivity as:

(1) $$k_{\text{rock}}=\left(\Delta m\times L\right)/\left(\Delta t\times A\times P\right)\;\text{kg}\;s^{-1}\;m^{-1}\;{\text{MPa}}^{-1}$$

where ∆ *m* is the water mass collected over a time interval∆ *t*, *L* is the sample length, *A* is the cross‐sectional area of the sample and P is the pressure applied in the pressure chamber.

### 2.2 Rock Porosity Measurements

Rock porosity was estimated by first immersing the samples in distilled water and exposing them to −60 kPa vacuum pressure for 6 days that is, until no visible air bubbles were present on their surface even after gently shaking. After measuring their saturated mass, the samples were oven‐dried at 70°C for 7 days that is, until stable weight was reached in two consecutive daily measurements, and their dry mass was recorded. Porosity was then calculated as the ratio of the water volume in the rock pores (obtained subtracting the dry weight from the wet weight of the rock samples) to the total volume of the cylinder.

Rock porosity was also assessed using 3D X‐ray Micro Computed Tomography scans of small rock fragments at the SYRMEP beamline of the Elettra Synchrotron Trieste (Trieste, Italy). We used the Propagation Based phase contrast modality and utilised an Orca Flash 4.0 SCMOS with a 17 μm GGG scintillator as detector. Settings included a detector distance of 15 cm, pixel size of 0.9 μm, white beam mode with a 1.0 mm Silica filter, yielding an average X‐ray energy of 22 keV. Throughout the 180° sample rotation, 1800 projections were captured. Slices were reconstructed using the SYRMEP Tomo Project software (Brun et al. 2015). Image enhancement involved a phase retrieval pre‐processing algorithm (Paganin et al. 2002) before applying the Filtered Back Projection. After denoising with Avizo's “Nonlocal Mean Filter” (Thermo Fisher Scientific 2019), images were sharpened with “Unsharp Masking” for clarity, then segmented to visualise air pores and the rock matrix. Porosity indices were calculated in cubes of 400 × 400 × 400 pixels (*n* = 3 for both Breccia and Dolostone), which were randomly extracted from the original scans. Images were binarized to segment air pores and the rock matrix using a custom script in Python software. Specifically, for each cube, one threshold value for binarization was selected by visually inspecting four slices (one slice every 100 slices). The threshold value resulting in the most accurate air pores and rock matrix segmentation was then used to binarize all the slices in each cube. The following porosity indices were calculated on segmented cubes using Pore3D software (Brun et al. 2010): air‐filled porosity (Porosity, %), connectivity density (CD, mm^−^^3^), coordination number and pores width (mm).

Porosity was calculated as:

(2) Porosity=(V_pores/V)×100%

where *V*_pores represents the volume occupied by air pores within the cube and *V* is the total cube volume. Connectivity density represents the number of redundant connections between pores normalised by V and was calculated as:

(3) $$\text{Connectivity}\;\text{density}\;=\left(1-\left(n-b\right)\right)/V\;{\text{mm}}^{-3}$$

where *n* is the number of pores and *b* is the number of pore‐to‐pore connections. Coordination number is the average number of branches that spread out of each pore. Pores width was computed as the median diameter of the maximal inscribed sphere in each pore.

### 2.3 Root Hydraulics

Initially, daily water loss of the different plant groups was assessed by measuring the difference in weight of samples (pot + plant) over a 24 h time interval, with the initial weight being measured 1 h after the last irrigation to pot capacity. Height and diameter of plants were also measured, and relative growth rates in height (RGRH) and in diameter (RGRD) calculated with reference to the values recorded at the beginning of the experiment (see above). Each experimental group (S, B, D) was then split into two sub‐groups. One group was maintained under optimal irrigation conditions (well‐watered samples: SWW, BWW, DWW, *N* = 7). A second group was subjected to water stress by irrigating plants with only 30% of daily water consumption (as estimated in controls, see above) for 7 days, and then suspending irrigation on Day 8 (stressed samples: SS, BS, DS, *N* = 6). This treatment caused a progressive reduction of Predawn leaf water potential to about −3 MPa, corresponding to the water potential at turgor loss point (Nardini et al. 2003, Tomasella et al. 2019) and to the onset of critical xylem embolism levels in *F. ornus* (Petit et al. 2016, Natale et al. 2023). Predawn leaf water potential was measured using a Scholander pressure chamber (mod. 1505D, PMS Instrument Company, Albany, OR, USA). Measurements were done on leaves sampled between 7.00 and 8.00 a.m., collected from plants enclosed in black plastic bags 12 h before sampling.

At the end of the drought treatment, root hydraulic conductance (K) was measured in well irrigated and drought stressed plants from the three experimental groups. Measurements were performed with a High‐Pressure Flow Meter (HPFM, Tyree et al. 1995) following procedures detailed in Nardini and Tyree (1999). After each measurement, the root system was carefully cleaned from soil and rock fragments, and placed on a glass tray filled with a film of water laying over a flatbed scanner. The total length (*L*_root_) and surface area (*A*_root_) of the root system was calculated using the RhizoVision software V2.0.2 (Seethepalli et al. 2021). Leaves were also collected and placed on a scanner to calculate total leaf surface area (*A*_leaf_) via ImageJ (Schindelin et al. 2012). Finally, above‐ and below‐ground dry biomass was measured after oven‐drying at 70°C for 24 h.

Root specific hydraulic conductivity (*k*_root_) and leaf area‐normalised root hydraulic conductance (*K*_root_) were calculated as:

(4) $$k_{\text{root}}=K\;\times \;(L_{\text{root}}/A_{\text{root}})\;\text{kg}\;s^{-1}\;m^{-1}\;{\text{MPa}}^{-1}$$

(5) $$K_{\text{root}}=K/A_{\text{leaf}}\;\text{kg}\;s^{-1}\;m^{-2}\;{\text{MPa}}^{-1}$$

Root‐to‐shoot ratio was calculated as the ratio between above‐ground (leaves + stems) and below‐ground (roots) biomass.

To test for possible effects of different soil‐rock mixtures and drought stress on root vitality (Savi et al. 2016), an electrolyte leakage test was carried out on root samples collected from a total of 9 drought‐stressed plants (three per group) following Tomasella et al. (2022). After cleaning the roots, 100 mg of fresh material were rinsed with deionized water and placed in a 1.5 mL Eppendorf tube filled with 1.5 mL of mQ water. The tube was shaken for 60 min, then refilled with 1 mL of mQ water and shaken for another 30 min. Initial electrical conductivity (EC_i_ μS cm^−^^1^) was recorded using a conductivity metre (LAQUAtwin‐EC‐11, Horiba Ltd. Kyoto, Japan). The tubes were then cycled three times between freezing in liquid nitrogen for 1 min and thawing at laboratory temperature for 30 min. Final electrical conductivity (EC_f_) was then measured. The test was replicated three times for each plant and the Relative Electrolyte Leakage (REL, %) was calculated as:

(6) $$\text{REL}\;=\left({\text{EC}}_{i}/{\text{EC}}_{f}\right)\times 100\%$$

### 2.4 Water Exchange Model

Following the analysis of the hydraulic properties of soil, rocks and roots, we developed a water flow model to examine the impact of soil and rock hydraulic properties, and rock‐soil‐root contact on rock‐to‐root water transfer.

**Figure 1:**
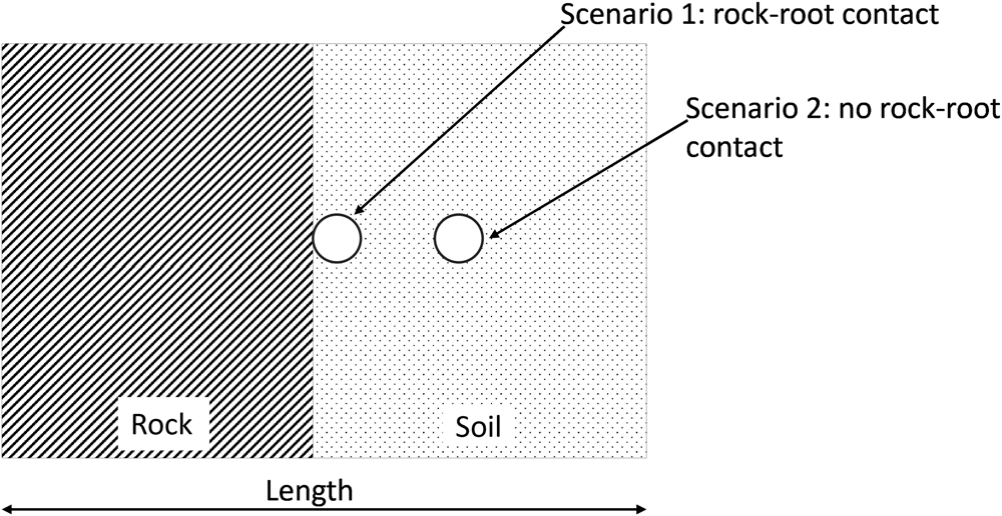
Schematic representation of the rock‐soil‐root system used in the model, with a total area of 9 mm², a root diameter of 1 mm, and a rock content of 40%. The model evaluates two scenarios: (1) root in contact with rock and (2) root not in contact with rock. The numerical solver employs the bimodal van Genuchten‐Mualem formulation (Durner et al. 1999) to assess water exchange efficiency across different spatial configurations of the rock‐soil‐root system.

Effective mechanistic modelling of water flow in soils requires quantitative evaluations of water content and pressure changes over time and space (Orgogozo et al. 2014). Hence, the most widely adopted approach involves numerically solving the three‐dimensional Richards equation in the relevant domain (Richards 1931). To this purpose, we used the RichardsFOAM (Orgogozo 2022) solver from the OpenFOAM software (Jasak 2009). The simplified system was represented as a 2D rectangle, with dimensions based on estimated Root Length Density (RLD) measured in potted plants when dividing *L*_root_ by the total pot volume, yielding RLD = 12 cm cm^−^^3^ and culminating in a 9 mm^2^ rectangle containing a single root with a diameter of 1 mm (Figure 1). To ensure that the results were independent of grid resolution, a grid convergence study was conducted using the Grid Convergence Index (GCI) method. Grid refinement, aimed at reducing discretization errors by increasing mesh resolution, was applied. After confirming grid convergence through the GCI analysis (see Supporting Information S1: Table S2), a final mesh size of 100 × 100 µm was selected for the simulations. Both high and low porosity rock types were included, occupying about 40% of the entire volume, the remaining being occupied by soil. We simulated two possible scenarios where the root was in contact or not with the rock surface.

Our solver implementation incorporated the bimodal van Genuchten‐Mualem formulation (Durner et al. 1999). Hydraulic parameters for the red soil and the rocks (B and D) were derived as outlined previously, along with water retention curves measured by Nardini et al. (2021). The hydraulic conductivity of the root was obtained as described above. We imposed the following boundary conditions. The rectangle's sides had a zero‐flux (Von Neumann) boundary condition, while a fixed water potential was set at the root endodermis (Dirichlet condition). The water potential range explored spanned from 0 (at saturation) to −1.5 MPa (conventional permanent wilting point). The simulation was run for a fixed duration, consistent across all scenarios, determined based on the system's stability at −1.5 MPa. The outcomes were processed using ParaView 5.11 (Ahrens, Geveci, and Law 2005).

### 2.5 Statistical Analysis

Statistical analyses were performed using R software (v. 4.3.3, R Core Team 2023). Rock saturated hydraulic conductivity and porosity differences were evaluated with Wilcoxon rank‐sum test as they were non‐normally distributed, using “wilcox. test” function in *stats* R package. Differences of greenhouse experimental data among *F. ornus* saplings grown in red soil (S) and mixture of red soil and Breccia (B) or Dolostone (D) were assessed with parametric and nonparametric (i.e, Kruskal‐Wallis) one‐way analysis of variance tests, using “aov” and “Kruskal. test” functions, respectively, from the *stats* R package. Post‐hoc analysis employed Dunn pairwise tests with “dunnTest” function in R package (Ogle et al. 2023). Differences of porosity indices based on 3D X‐ray Micro Computed Tomography scans between Breccia (B) and Dolostone (D) rock fragments were assessed with *t*‐tests, using “t.test” function in *stats* R package.

## 3 Results

### 3.1 Hydraulic Properties of Soil and Rocks

Water retention and unsaturated hydraulic conductivity curves, derived on HYPROP and WP4C measurements, are shown in Supporting Information S1: Figure S1. The falling head method based on KSAT yielded an average soil saturated hydraulic conductivity of 4.48 ± 0.33 kg m^−^^1^ s^−^^1^ MPa^−^^1^. The plant available water content, measured as the difference between the water content at field capacity and the water content at a soil water potential of −1.5 MPa (permanent wilting point) was 0.11 g g^−^^1^.

**Figure 2:**
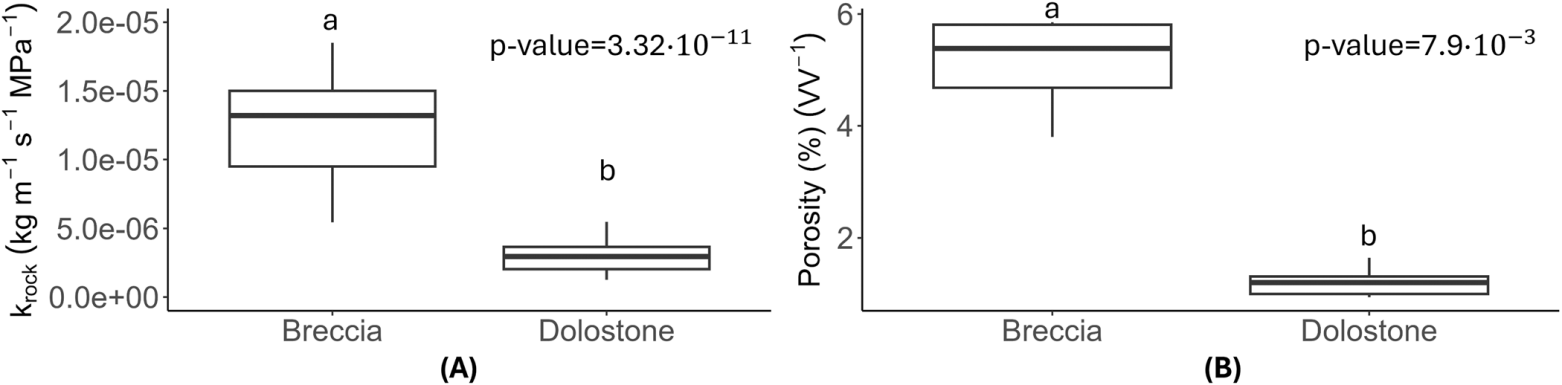
Median values, 25% and 75% percentiles of (A) rock hydraulic conductivity (*k*_rock_) and (B) primary porosity for Breccia (*N* = 5) and Dolostone (*N* = 5). Different letters indicate statistically significant differences between the two rock types (*p* < 0.05, Wilcoxon rank sum test). Corresponding p‐values are reported.

Rock saturated hydraulic conductivity (k_rock_) was significantly higher in Breccia samples, averaging 6.8 ± 4.1 × 10^−^^5^ kg m^−^^1^ s^−^^1^ MPa^−^^1^, than in Dolostone ones, where the same parameter was 2.9 ± 0.3 × 10^−^^6 ^kg m^−^^1^ s^−^^1^ MPa^−^^1^ (Figure 2a). Breccia samples exhibited greater variability in terms of k_rock_ as also reflected by the variability of primary porosity (5.4% ± 1%), when compared to Dolostone samples (1.2% ± 0.3%, Figure 2b).

**Figure 3:**
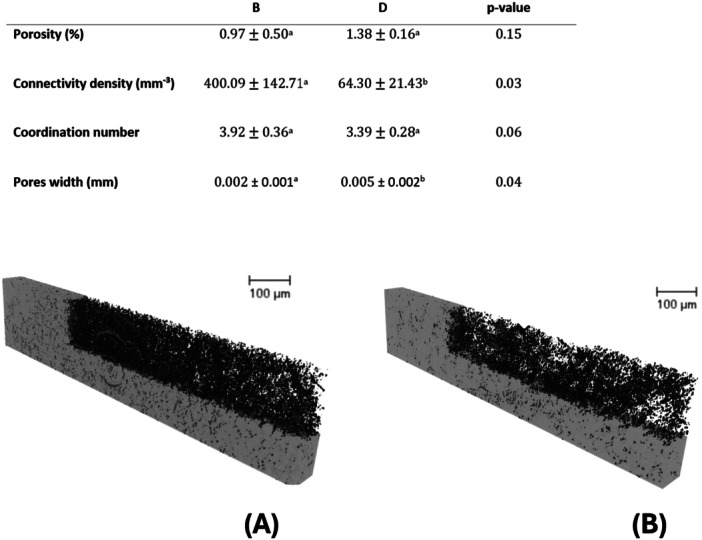
3D Micro‐CT reconstructions of Breccia (A) and Dolostone (B) rock samples, with corresponding parameters derived from 3D image analysis (see the upper table). Values are presented as mean ± SD (*N* = 3). Statistically significant differences between groups (*p* < 0.05, *t*‐test) are indicated by different letters. Pore volumes are shown in black, and the rock matrix is depicted in grey. The acronyms (B) and (D) in the upper table represent Breccia and Dolostone, respectively.

X‐ray Micro‐CT analyses further revealed higher pores connectivity in Breccia than in Dolostone samples (Figure 3). Specifically, connectivity was one order of magnitude higher in Breccia than in Dolostone, while Porosity was not significantly different, in contrast with results obtained with direct measurements of this parameter (Figure 2b). Coordination number was not statistically different between the two rock types, while Dolostone rock samples had significantly higher pores width than Breccia ones (Figure 3).

### 3.2 Plant Growth and Root Hydraulics

Mean values and standard deviations of RGRH, RGRD and Root/Shoot ratio as measured in saplings grown in different mixtures of soil and rocks are presented in Table 1. The RGRH and Leaf area of plants grown in pure soil were significantly higher than those of plants grown in soil/rock mixtures. RGRD and Root/Shoot ratio were not significantly different among experimental groups.

**Table 1:** Relative growth rates in terms of plant height (RGRH) or basal diameter (RGRD), root/shoot biomass ratio, and total leaf surface area measured in saplings grown in different soil/rock mixtures. S: soil only; B: mix of soil and Breccia; D: mix of soil and Dolostone. Values are given as mean ± SD (N = 13). Different letters indicate statistically significant differences (*p* < 0.05, Kruskal‐Wallis test and Dunn test) among groups.

	*S*	*B*	*D*
RGRH (%)	205 ± 68^*a*^	$153\pm 51^{b}$	$162\pm 72^{b}$
RGRD (%)	$14\pm 7^{a}$	$14\pm 11^{a}$	$13\pm 6^{a}$
Root/Shoot (g g^−1^)	$0.62\pm 0.20^{a}$	$0.62\pm 0.19^{a}$	$0.62\pm 0.38^{a}$
Leaf area (dm^2^)	6.9 ± 0.2^*a*^	4.4 ± 0.9^*b*^	4.8 ± 0.8^*b*^

**Figure 4:**
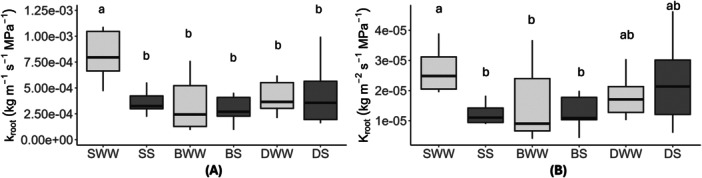
Boxplots of root hydraulic conductivity (A, *k*_root_) and leaf‐area normalised root hydraulic conductance (B, *K*_root_) measured in saplings exposed to different experimental conditions: Soil Well Watered (SWW), Soil Stressed (SS), Breccia Well Watered (BWW), Breccia Stressed (BS), Dolostone Well Watered (DWW), and Dolostone Stressed (DS). Light grey boxes represent well‐watered samples, and dark grey ones represent water‐stressed samples. Stressed samples had *N* = 6, and well‐watered samples had *N* = 7. Different letters indicate statistically significant differences among groups (*p* < 0.05). Kruskal‐Wallis followed by Dunn's pairwise test was conducted on all treatments for both (A) and (B).

Figure 4 reports values of root hydraulic conductivity (*k*_root_) and leaf‐area‐normalised root hydraulic conductance (*K*_root_) measured in the different experimental groups under both well‐watered and drought conditions. No significant differences were observed between treatments or groups, except for the well‐watered S samples (SWW) that exhibited the highest *k*_root_, while no significant differences were detected between the other treatments. A similar tendency was observed for *K*_root_, where SWW had significantly higher values compared to SS and BS, while DWW and DS were not significantly different from any other treatment (SWW, SS, BS, or BWW). Compared to values recorded in SWW plants, both parameters decreased by about 50% in the other groups. Overall, *k*_root_ ranged between 7.5 and 2.5 × 10^−^^4 ^kg m^−^^1^ s^−^^1^ MPa^−^^1^, in well‐watered plants grown in soil and all the other groups, respectively.

Relative electrolyte leakage mean values and standard deviations are presented in Supporting Information S1: Table S1. No statistical differences emerged between S, B and D water‐stressed samples.

### 3.3 Water Exchange Model

**Figure 5:**
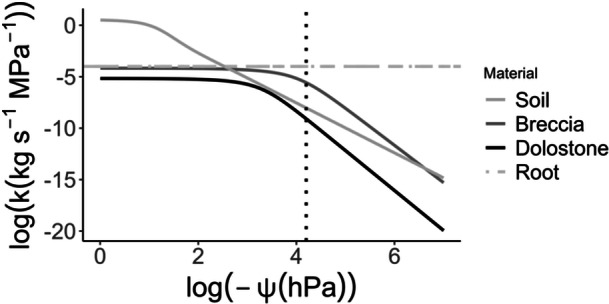
Log transformed unsaturated hydraulic conductivity (*k*) as a function of water potential (ψ) in Soil, Breccia, Dolostone, and in the Root. Water potential values considered in the model ranged from 0 to −1.5 MPa. The dotted line at 4.2 correspond to a water potential of −1.5 MPa.

**Figure 6:**
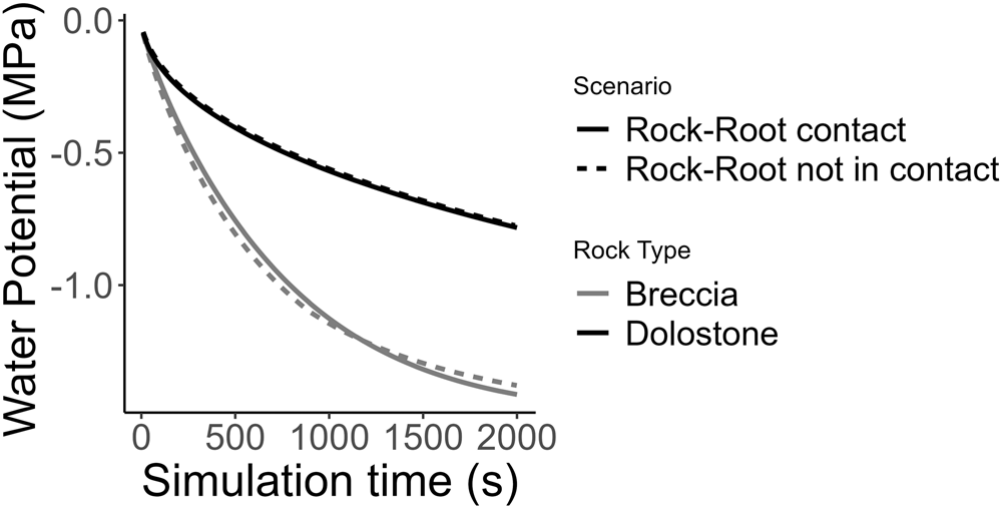
Maximum water potential of soil and rock as a function of simulation time in a system with Dolostone or Breccia, for both root‐rock contact and non‐contact scenarios. The model used the bimodal van Genuchten‐Mualem formulation (Durner et al. 1999) to assess how soil and rock hydraulic properties, as well as root‐rock contact, influence water transfer to roots. Rocks occupied ~40% of the volume, with soil filling the rest. A 100 × 100 µm mesh size was used, and simulations explored the dynamics of water potential over time, ranging from 0 to −1.5 MPa. Boundary conditions included zero‐flux at the sides and fixed water potential at the root endodermis.

**Figure 7:**
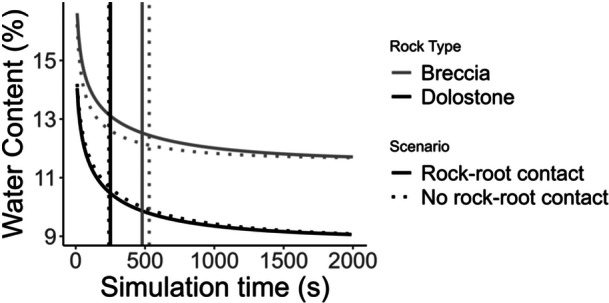
Average water content as a function of the simulation time in the model system with Breccia or with Dolostone. Vertical lines indicate the simulation time corresponding to the 25% decrease of initial water content for both rock types and for the root‐rock in contact (solid line) and root‐rock not in contact (dotted line) scenarios.

In the water potential range set in the model, the soil unsaturated hydraulic conductivity initially decreases more rapidly than in Breccia rock matrix, and remains above that of Dolostone for the whole water potential range tested (Figure 5). Numerical simulations of water flow in the rock‐soil‐root system indicate that the water potential reaches the permanent wilting point within 40 min in the soil‐Breccia system, whereas it decreases slowly in the soil‐Dolostone system (Figure 6). For the soil‐Breccia system, the water potential initially decreases slightly faster when there is no root‐rock contact. However, as the system approaches the wilting point, the scenario with root‐rock contact surpasses the no‐contact scenario in terms of the speed of water potential decrease. Similarly, the rock water content declines more rapidly in the Breccia system compared to the Dolostone one, particularly when direct root‐rock contact is assumed (Figure 7). The time required by the Breccia system to undergo a 25% decrease in water content is almost double compared to the Dolostone system, with slight differences based on the eventual presence of direct root‐rock contact (Figure 7). As the system dries and reaches the permanent wilting point, the soil water content drops to about 15%, consistently with the soil water retention.

## 4 Discussion

Our study aimed at testing different hypotheses on the potential efficiency of rock‐to‐root water transfer under different levels of water stress at soil and plant level. Our first aim was to compare rock properties with those of a typical karstic red soil. The combination of the HYPROP system, which has limitations at both extremes of the soil moisture spectrum, with complementary methods such as KSAT and WP4C enabled a precise fitting of the unsaturated soil hydraulic conductivity and water retention curves. Our data for the soil under study yielded a plant available water content (AWC) of 0.11 g g^−^^1^, as derived from the difference between the water content at field capacity and at the permanent wilting point. When comparing the AWC for the same water potential range considered by Nardini et al. (2021) for the same red soil, our data align with these previously reported values, as well as with AWC reported in the literature for other Karst soils (Savi et al. 2019)

The next phase involved assessing the hydraulic properties of the rock types considered in the study and investigated by Nardini et al. (2021), that is, Breccia and Dolostone. The k_rock_ of B was one order of magnitude higher (6.8 ± 4.1 × 10^−^^5 ^kg s^−1^ m^−^^1^ MPa^−^^1^) than that recorded for D (2.9 ± 0.3 × 10^−6 ^kg s^−^^1^ m^−^^1^ MPa^−^^1^). Both values are within previously reported ranges for limestone (Boving and Grathwohl 2001, Pulido‐Bosch et al. 2004), thus supporting the validity of our simple experimental method for measuring rock hydraulics that might be used for future studies in different rock types from different ecological contexts. Indeed, our measurements are somewhat novel with respect to the scale at which they were conducted, and because they were designed to provide estimates of rock hydraulics parameters easily comparable to those more commonly used in the study of plant hydraulics.

The much higher k_rock_ values of B compared to D indicate more efficient water flow through the Breccia matrix, likely due to its higher pore connectivity. This was supported by the porosity measurements based on weight difference between water‐saturated and oven‐dried rock samples, as well as by the connectivity density index derived on microCT‐based 3D reconstructions of the porous network of small rock samples. Dolostone is a very compact rock which predominantly consists of the mineral dolomite CaMg(CO_3_)_2_, with a varying yet significant proportion of calcium carbonate CaCO_3_. In contrast, Breccia is a carbonate limestone rock mainly composed of CaCO_3_ and characterised by large, angular fragments (Pettijohn 1957). These differences in chemical and structural composition likely account for the different hydraulic properties of the two rock types recorded in this study, but also for the larger variability in terms of *k*_rock_ and primary porosity observed across different Breccia samples. This large variability probably explains also the lack of significant differences between rock types in terms of porosity when assessed via microCT in a limited number of samples, due to time‐ and cost‐related constraints. When combining the higher *k*_rock_ of B compared to D with parallel differences in AWC previously reported for these two rock types (Nardini et al. 2021), it can be concluded that B rocks not only store more moisture in the pore space, but also can potentially deliver this water more efficiently to the surrounding soil and/or to eventual roots in close proximity, provided a downward gradient in water potential between these different compartments, thus supporting our working hypothesis. On the other hand, the low AWC and very low saturated hydraulic conductivity of D rocks, which are indicative of its limited ability to store and transfer water even under optimal conditions, suggest that this rock type might not represent a significant and useful water reservoir for plants. These aspects might explain why *F. ornus* plants growing on D bedrock experience more water stress compared to plants of the same species thriving on B bedrock (Nardini et al. 2021).

Hydraulic measurements performed on potted plants revealed significant impact of both water stress and the presence of rock fragments on root hydraulics, and allowed us to compare the hydraulic efficiencies of root, rocks and soil. We expected *k*_root_ to be higher than *k*_rock_ under well‐watered conditions, but also to decline under drought thus approaching *k*_rock_ when water in the soil becomes limiting. Values of *K*_root_ found in our study (Figure 4) aligned with those previously reported for saplings of different woody plants (e.g., Lo Gullo et al. 1998, Nardini and Tyree 1999, Nardini, Lo Gullo, and Salleo 1999, Nardini et al. 2006). Under saturated conditions, *k*_root_ was four orders of magnitude lower than k_soil_, yet two to three orders of magnitude higher than *k*_rock_ of Breccia and Dolostone, respectively (Figure 4). Hence, under saturated conditions *k*_root_ was by far the limiting factor for water uptake from the soil, while rock moisture was probably delivered at excessively low rates to represent a significant contribution to total transpiration of *F. ornus* plants (Nardini et al. 2003). However, under drought conditions the hydraulic conductivities of soil and rock significantly decreased and became similar, while k_root_ dropped by about 50% so that root hydraulics ceased to represent the main limiting factor for water absorption.

Root hydraulic conductivity measurements revealed no major differences between different treatments in terms of rock‐soil mixtures and irrigation levels, with the only exception represented by samples grown in well irrigated soil with no rock fragments, which exhibited 50% higher *k*_root_ than the other treatments. Both drought stress and the presence of rock fragments reduced root hydraulic efficiency, although leaf‐based root hydraulic conductance was similar in all experimental groups as an effect of the reduction of leaf surface area in B and D samples compared to S ones. Interestingly, k_root_ was reduced in plants grown in mixed soil/rock substrates compared to only soil, regardless of water status, suggesting that the presence of rock fragments lead per se to hydraulic limitations. This might be a consequence of reduced total available water (AWC) in pots with a significant volume of rock fragments, although this does not explain the reduced *k*_root_ in well‐irrigated plants. Relative electrolyte leakage measurements showed no significant cell membrane damage due to the presence of rock fragments, regardless of eventual water stress. Hence, differences in *k*_root_ between well‐irrigated S, B and D plants, as well as the similarity of *k*_root_ of well irrigated B and D plants with that of drought‐stressed S plants suggest a convergent acclimation process of the root system in response to both the presence of rocks and the reduction of substrate water content. Indeed, the presence of rocks in the substrate imposes mechanical impedance, limiting spatial expansion of the root system compared to homogenous soil environments, where roots can freely extend in any direction. The mechanical impedance of substrates is well known to induce several morphological, anatomical and physiological adjustments in the root system (Potocka and Szymanowska‐Pułka 2018, Jacobsen et al. 2021) resembling responses of root growth to water limitations (Bengough et al. 2011), and possibly leading to a convergent impact on root hydraulic properties. Hence, the presence of rock fragments in the soil might mimic the effects of drought stress on root systems, possibly because of similar signalling pathways initiated by ABA and ethylene accumulation (Liang et al. 1999, Jacobsen et al. 2021), finally leading to a decline in *k*_root_ (Miniussi et al. 2015, Tataranni et al. 2015). When compensated by a reduction in leaf surface area (Zhang et al. 2024), the leaf‐level K_root_ might remain unaltered despite reduction of root hydraulic efficiency, as indeed observed in our study.

Based on the above, we tested our last hypothesis, that is, that rock‐to‐root water delivery is only possible under specific conditions. Indeed, following the characterisation of the hydraulic properties of single components of the rock‐soil‐root system, the critical next step was to elucidate the possible dynamics of water flow within this system and quantify the possible contribution of the rock matrix to the water volumes available for root uptake. The simple 2D single root model designed to simulate moisture redistribution in a mixed rock/soil substrate under drought conditions allowed us to gain interesting insights. Overall, simulations confirmed our hypothesis that the higher hydraulic conductivity of Breccia facilitates root absorption of water from the rock matrix, even without direct root‐rock contact. Conversely, the water stored within D rocks appears to be less accessible to roots, indicating that it might not significantly contribute to water supply to the plant. These findings are consistent with the beneficial effects of Breccia bedrock on plant water status of *F. ornus* trees in the field under summer drought, an effect that was lacking in trees of the same species growing over Dolostone bedrock (Nardini et al. 2021). Our simulations assumed direct root‐rock contact as a best‐case scenario, but it is possible that rock water uptake by plants is mediated by mycorrhizal hyphae that might directly penetrate the primary pores of the rock (Schwinning 2020, Nardini, Tomasella, and Di Bert 2024). It would be interesting to run simulations including this possible hydraulic pathway, provided the availability of accurate estimates of hyphal hydraulic conductivity (Eamus et al. 1985) and spatial analysis of their eventual presence, abundance and distribution in the rock matrix (Burford, Kierans, and Gadd 2003).

Research on models of root water uptake that account not only for soil and root hydraulic properties but also consider the contribution of rocks at such detailed scales is still very limited. Our model, despite its simplicity, provides support to field observations of better water status for plants growing over porous bedrock compared to more compact one (Nardini et al. 2021), and opens new directions in the study of below‐ground plant water relations in rock‐dominated landscapes. We call for further model implementations considering more intricate and 3D scenarios of rock‐root‐soil interactions, that might further help to recognise water stored in bedrock as an important resource sustaining tree hydration during drought periods.

## Supporting information

Supporting information.

## Data Availability

The data that support the findings of this study are available from the corresponding author upon reasonable request.

## References

[pce15369-bib-0001] Adams, H. D. , G. A. Barron‐Gafford , R. L. Minor , et al. 2017. “Temperature Response Surfaces for Mortality Risk of Tree Species With Future Drought.” Environmental Research Letters 12: 115014.

[pce15369-bib-0002] Ahrens, J. , B. Geveci , and C. Law . 2005. “ParaView: An End‐User Tool for Large Data Visualization.” In Visualization Handbook. Elsevier.

[pce15369-bib-0003] Allen, C. D. , A. K. Macalady , H. Chenchouni , et al. 2010. “A Global Overview of Drought and Heat‐Induced Tree Mortality Reveals Emerging Climate Change Risks for Forests.” Forest Ecology and Management 259: 660–684.

[pce15369-bib-0004] Baguskas, S. A. , S. H. Peterson , B. Bookhagen , and C. J. Still . 2014. “Evaluating Spatial Patterns of Drought‐Induced Tree Mortality in a Coastal California Pine Forest.” Forest Ecology and Management 315: 43–53.

[pce15369-bib-0005] Bengough, A. G. , B. M. McKenzie , P. D. Hallett , and T. A. Valentine . 2011. “Root Elongation, Water Stress, and Mechanical Impedance: A Review of Limiting Stresses and Beneficial Root Tip Traits.” Journal of Experimental Botany 62: 59–68. 21118824 10.1093/jxb/erq350

[pce15369-bib-0006] Boving, T. B. , and P. Grathwohl . 2001. “Tracer Diffusion Coefficients in Sedimentary Rocks: Correlation to Porosity and Hydraulic Conductivity.” Journal of Contaminant Hydrology 53: 85–100. 11816996 10.1016/s0169-7722(01)00138-3

[pce15369-bib-0007] Brun, F. , L. Mancini , P. Kasae , S. Favretto , D. Dreossi , and G. Tromba . 2010. “Pore3D: A Software Library for Quantitative Analysis of Porous Media.” Nuclear Instruments and Methods in Physics Research Section A: Accelerators, Spectrometers, Detectors and Associated Equipment 615: 326–332.

[pce15369-bib-0008] Brun, F. , S. Pacilè , A. Accardo , et al. 2015. “Enhanced and Flexible Software Tools for X‐Ray Computed Tomography at the Italian Synchrotron Radiation Facility Elettra.” Fundamenta Informaticae 141: 233–243.

[pce15369-bib-0009] Burford, E. P. , M. Kierans , and G. M. Gadd . 2003. “Geomycology: Fungi in Mineral Substrata.” Mycologist 17: 98–107.

[pce15369-bib-0010] Chitra‐Tarak, R. , L. Ruiz , H. S. Dattaraja , et al. 2018. “The Roots of the Drought: Hydrology and Water Uptake Strategies Mediate Forest‐Wide Demographic Response to Precipitation.” Journal of Ecology 106: 1495–1507.

[pce15369-bib-0011] Choat, B. , T. J. Brodribb , C. R. Brodersen , R. A. Duursma , R. López , and B. E. Medlyn . 2018. “Triggers of Tree Mortality under Drought.” Nature 558: 531–539. 29950621 10.1038/s41586-018-0240-x

[pce15369-bib-0012] Choat, B. , S. Jansen , T. J. Brodribb , et al. 2012. “Global Convergence in the Vulnerability of Forests to Drought.” Nature 491: 752–755. 23172141 10.1038/nature11688

[pce15369-bib-0013] Diouf, O. C. , L. Weihermüller , M. Diedhiou , et al. 2020. “Modelling Groundwater Evapotranspiration in a Shallow Aquifer in a Semi‐Arid Environment.” Journal of Hydrology 587: 124967.

[pce15369-bib-0014] Durner, W. , E. Priesack , H. J. Vogel , and T. Zurmühl . 1999. “Determination of Parameters for Flexible Hydraulic Functions by Inverse Modeling.” In Characterization and Measurement of the Hydraulic Properties of Unsaturated Porous Media, edited by M. T. van Genuchten , F. J. Leij , and L. Wu , 817–829. Riverside (CA): University of California.

[pce15369-bib-0015] Eamus, D. , W. Thompson , J. W. G. Cairney , and D. H. Jennings . 1985. “Internal Structure and Hydraulic Conductivity of Basidiomycete Translocating Organs.” Journal of Experimental Botany 36: 1110–1116.

[pce15369-bib-0016] Graham, R. C. , M. A. Anderson , P. D. Sternberg , K. R. Tice , and P. J. Schoeneberger . 1997. “Morphology, Porosity, and Hydraulic Conductivity of Weathered Granitic Bedrock and Overlying Soils.” Soil Science Society of America Journal 61: 516–522.

[pce15369-bib-0017] Hahm, W. J. , D. M. Rempe , D. N. Dralle , T. E. Dawson , and W. E. Dietrich . 2020. “Oak Transpiration Drawn From the Weathered Bedrock Vadose Zone in the Summer Dry Season.” Water Resources Research 56: e2020WR027419.

[pce15369-bib-0018] Hartmann, H. , A. Bastos , A. J. Das , et al. 2022. “Climate Change Risks to Global Forest Health: Emergence of Unexpected Events of Elevated Tree Mortality Worldwide.” Annual Review of Plant Biology 73: 673–702. 10.1146/annurev-arplant-102820-012804 35231182

[pce15369-bib-0019] Hellmers, H. , J. S. Horton , G. Juhren , and J. O'Keefe . 1955. “Root Systems of Some Chaparral Plants in Southern California.” Ecology 36: 667–678.

[pce15369-bib-0020] Jacobsen, A. G. R. , G. Jervis , J. Xu , J. F. Topping , and K. Lindsey . 2021. “Root Growth Responses to Mechanical Impedance Are Regulated by a Network of ROS, Ethylene and Auxin Signalling in *Arabidopsis* .” New Phytologist 231: 225–242. 33428776 10.1111/nph.17180 PMC8651006

[pce15369-bib-0021] Jasak, H. 2009. “Openfoam: Open Source CFD in Research and Industry.” International Journal of Naval Architecture and Ocean Engineering 1: 89–94.

[pce15369-bib-0022] Johnson, D. M. , J. C. Domec , Z. Carter Berry , et al. 2018. “Co‐Occurring Woody Species Have Diverse Hydraulic Strategies and Mortality Rates During an Extreme Drought.” Plant, Cell & Environment 41: 576–588. 10.1111/pce.13121 29314069

[pce15369-bib-0023] De Jong van Lier, Q. , M. L. A. de Melo , and E. A. R. Pinheiro . 2024. “Stochastic Analysis of Plant Available Water Estimates and Soil Water Balance Components Simulated by a Hydrological Model.” Vadose Zone Journal 23: e20306.

[pce15369-bib-0024] Jurkovšek, B. , S. Biolchi , S. Furlani , et al. 2016. “Geology of the Classical Karst Region (SW Slovenia–NE Italy).” Journal of Maps 12: 352–362.

[pce15369-bib-0026] Klein, T. , M. J. B. Zeppel , W. R. L. Anderegg , et al. 2018. “Xylem Embolism Refilling and Resilience against Drought‐Induced Mortality in Woody Plants: Processes and Trade‐Offs.” Ecological Research 33: 839–855.

[pce15369-bib-0027] Korboulewsky, N. , M. Tétégan , A. Samouelian , and I. Cousin . 2020. “Plants Use Water in the Pores of Rock Fragments During Drought.” Plant and Soil 454: 35–47.

[pce15369-bib-0028] Liang, J. , J. Zhang , G. Y. S. Chan , and M. H. Wong . 1999. “Can Differences in Root Responses to Soil Drying and Compaction Explain Differences in Performance of Trees Growing on Landfill Sites?” Tree Physiology 19: 619–624. 12651537 10.1093/treephys/19.9.619

[pce15369-bib-0029] Lo Gullo, M. A. , A. Nardini , S. Salleo , and M. T. Tyree . 1998. “Changes in Root Hydraulic Conductance (K_R_) of *Olea Oleaster* Seedlings Following Drought Stress and Irrigation.” New Phytologist 140: 25–31.

[pce15369-bib-0030] MacGregor, S. D. , and T. G. O'Connor . 2002. “Patch Dieback Ofcolophospermum Mopanein a Dysfunctional Semi‐Arid African Savanna.” Austral Ecology 27: 385–395.

[pce15369-bib-0031] McCormick, E. L. , D. N. Dralle , W. J. Hahm , et al. 2021. “Widespread Woody Plant Use of Water Stored in Bedrock.” Nature 597: 225–229. 34497393 10.1038/s41586-021-03761-3

[pce15369-bib-0032] McDowell, N. G. , D. J. Beerling , D. D. Breshears , R. A. Fisher , K. F. Raffa , and M. Stitt . 2011. “The Interdependence of Mechanisms Underlying Climate‐Driven Vegetation Mortality.” Trends in Ecology & Evolution 26: 523–532. 21802765 10.1016/j.tree.2011.06.003

[pce15369-bib-0033] McDowell, N. G. , G. Sapes , A. Pivovaroff , et al. 2022. “Mechanisms of Woody‐Plant Mortality Under Rising Drought, CO_2_ and Vapour Pressure Deficit.” Nature Reviews Earth & Environment 3: 294–308.

[pce15369-bib-0034] Miniussi, M. , L. Del Terra , T. Savi , A. Pallavicini , and A. Nardini . 2015. “Aquaporins in *Coffea Arabica* L.: Identification, Expression, and Impacts on Plant Water Relations and Hydraulics.” Plant Physiology and Biochemistry 95: 92–102. 26241904 10.1016/j.plaphy.2015.07.024

[pce15369-bib-0035] Montaldo, N. , R. Corona , M. Curreli , S. Sirigu , L. Piroddi , and R. Oren . 2021. “Rock Water As a Key Resource for Patchy Ecosystems on Shallow Soils: Digging Deep Tree Clumps Subsidize Surrounding Surficial Grass.” Earth's Future 9: e2020EF001870.

[pce15369-bib-0036] Nardini, A. , M. Battistuzzo , and T. Savi . 2013. “Shoot Desiccation and Hydraulic Failure in Temperate Woody Angiosperms During an Extreme Summer Drought.” New Phytologist 200: 322–329. 23593942 10.1111/nph.12288

[pce15369-bib-0037] Nardini, A. , V. Casolo , A. Dal Borgo , et al. 2016. “Rooting Depth, Water Relations and Non‐Structural Carbohydrate Dynamics in Three Woody Angiosperms Differentially Affected by an Extreme Summer Drought.” Plant, Cell & Environment 39: 618–627. 10.1111/pce.12646 26437327

[pce15369-bib-0038] Nardini, A. , A. Gasco , F. Raimondo , et al. 2006. “Is Rootstock‐Induced Dwarfing in Olive an Effect of Reduced Plant Hydraulic Efficiency?” Tree Physiology 26: 1137–1144. 16740489 10.1093/treephys/26.9.1137

[pce15369-bib-0039] Nardini, A. , M. A. Lo Gullo , and S. Salleo . 1999. “Competitive Strategies for Water Availability in Two Mediterranean *Quercus* Species.” Plant, Cell & Environment 22: 109–116.

[pce15369-bib-0040] Nardini, A. , F. Petruzzellis , D. Marusig , et al. 2021. “Water ‘On the Rocks': a Summer Drink for Thirsty Trees.” New Phytologist 229: 199–212. 32772381 10.1111/nph.16859

[pce15369-bib-0041] Nardini, A. , S. Salleo , P. Trifilò , and M. A. Lo Gullo . 2003. “Water Relations and Hydraulic Characteristics of Three Woody Species Co‐Occurring in the Same Habitat.” Annals of Forest Science 60: 297–305.

[pce15369-bib-0042] Nardini, A. , T. Savi , P. Trifilò , and M. A. Lo Gullo . 2018. “Drought Stress and the Recovery From Xylem Embolism in Woody Plants.” Prog Bot 79: 197–231.

[pce15369-bib-0043] Nardini, A. , M. Tomasella , and S. Di Bert . 2024. “Bedrock: The Hidden Water Reservoir for Trees Challenged by Drought.” Trees 38: 1–11.

[pce15369-bib-0044] Nardini, A. , and M. T. Tyree . 1999. “Root and Shoot Hydraulic Conductance of Seven *Quercus* Species.” Annals of Forest Science 56: 371–377.

[pce15369-bib-0045] Natale, S. , M. Tomasella , S. Gargiulo , et al. 2023. “Stem Photosynthesis Contributes to Non‐Structural Carbohydrate Pool and Modulates Xylem Vulnerability to Embolism in *Fraxinus Ornus* L.” Environmental and Experimental Botany 210: 105315.

[pce15369-bib-0046] Ogle, D. H. , J. C. Doll , A. P. Wheeler , and A. Dinno 2023. FSA: Simple Fisheries Stock Assessment Methods. R Package Version 0.9.5. https://CRAN.R-project.org/package=FSA.

[pce15369-bib-0047] Orgogozo, L. 2022. “RichardsFoam3: A New Version of Richardsfoam for Continental Surfaces Hydrogeology Modelling.” Computer Physics Communications 270: 108182.

[pce15369-bib-0048] Orgogozo, L. , N. Renon , C. Soulaine , et al. 2014. “An Open Source Massively Parallel Solver for Richards Equation: Mechanistic Modelling of Water Fluxes at the Watershed Scale.” Computer Physics Communications 185: 3358–3371.

[pce15369-bib-0049] Paganin, D. , S. C. Mayo , T. E. Gureyev , P. R. Miller , and S. W. Wilkins . 2002. “Simultaneous Phase and Amplitude Extraction From a Single Defocused Image of a Homogeneous Object.” Journal of Microscopy 206: 33–40. 12000561 10.1046/j.1365-2818.2002.01010.x

[pce15369-bib-0050] Peters, A. , and W. Durner . 2008. “Simplified Evaporation Method for Determining Soil Hydraulic Properties.” Journal of Hydrology 356: 147–162.

[pce15369-bib-0051] Petit, G. , T. Savi , M. Consolini , T. Anfodillo , and A. Nardini . 2016. “Interplay of Growth Rate and Xylem Plasticity for Optimal Coordination of Carbon and Hydraulic Economies in *Fraxinus Ornus* Trees.” Tree Physiology 36: 1310–1319. 27587483 10.1093/treephys/tpw069

[pce15369-bib-0052] Petrucco, L. , A. Nardini , G. von Arx , M. Saurer , and P. Cherubini . 2017. “Isotope Signals and Anatomical Features in Tree Rings Suggest a Role for Hydraulic Strategies in Diffuse Drought‐Induced Die‐Back of *Pinus Nigra* .” Tree Physiology 37: 523–535. 28338978 10.1093/treephys/tpx031

[pce15369-bib-0053] Pettijohn, F. J. 1957. Sedimentary Rocks. 2nd Edition. New York: Harper.

[pce15369-bib-0054] Potocka, I. , and J. Szymanowska‐Pułka . 2018. “Morphological Responses of Plant Roots to Mechanical Stress.” Annali di Botanica 122: 711–723. 10.1093/aob/mcy010 PMC6215033 29471488

[pce15369-bib-0055] Pulido‐Bosch, A. , J. Motyka , P. Pulido‐Leboeuf , and S. Borczak . 2004. “Matrix Hydrodynamic Properties of Carbonate Rocks From the Betic Cordillera (Spain).” Hydrological Processes 18: 2893–2906.

[pce15369-bib-0056] Querejeta, J. I. , H. Estrada‐Medina , M. F. Allen , J. J. Jiménez‐Osornio , and R. Ruenes . 2006. “Utilization of Bedrock Water by *Brosimum Alicastrum* Trees Growing on Shallow Soil Atop Limestone in a Dry Tropical Climate.” Plant and Soil 287: 187–197.

[pce15369-bib-0057] Rempe, D. M. , and W. E. Dietrich . 2018. “Direct Observations of Rock Moisture, a Hidden Component of the Hydrologic Cycle.” Proceedings of the National Academy of Sciences 115: 2664–2669. 10.1073/pnas.1800141115 PMC5856562 29490920

[pce15369-bib-0058] Richards, L. A. 1931. “Capillary Conduction of Liquids Through Porous Mediums.” Physics 1: 318–333.

[pce15369-bib-0025] Rose, K. , R. Graham , and D. Parker. 2003. “Water Source Utilization by *Pinus jeffreyi* and *Arctostaphylos Patula* on Thin Soils Over Bedrock.” Oecologia 134: 46–54. 12647178 10.1007/s00442-002-1084-4

[pce15369-bib-0059] Savi, T. , A. Dal Borgo , V. L. Love , S. Andri , M. Tretiach , and A. Nardini . 2016. “Drought Versus Heat: What's the Major Constraint on Mediterranean Green Roof Plants?” Science of the Total Environment 566–567–567: 753–760. 10.1016/j.scitotenv.2016.05.100 27239718

[pce15369-bib-0060] Savi, T. , F. Petruzzellis , S. Martellos , et al. 2018. “Vineyard Water Relations in a Karstic Area: Deep Roots and Irrigation Management.” Agriculture, Ecosystems & Environment 263: 53–59.

[pce15369-bib-0061] Savi, T. , F. Petruzzellis , E. Moretti , et al. 2019. “Grapevine Water Relations and Rooting Depth in Karstic Soils.” Science of the Total Environment 692: 669–675. 31539975 10.1016/j.scitotenv.2019.07.096

[pce15369-bib-0062] Schindelin, J. , I. Arganda‐Carreras , E. Frise , et al. 2012. “Fiji: An Open‐Source Platform for Biological‐Image Analysis.” Nature Methods 9: 676–682. 22743772 10.1038/nmeth.2019 PMC3855844

[pce15369-bib-0063] Schoeman, J. L. , M. M. Kruger , and A. H. Loock . 1997. “Water‐Holding Capacity of Rock Fragments in Rehabilitated Open Cast Mine Soils.” S Afr Tydskr Plant Grond 14: 98–102.

[pce15369-bib-0064] Schwinning, S. 2010. “The Ecohydrology of Roots in Rocks.” Ecohydrology 3: 238–245.

[pce15369-bib-0065] Schwinning, S. 2020. “A Critical Question for the Critical Zone: How Do Plants Use Rock Water.” Plant and Soil 454: 49–56.

[pce15369-bib-0066] Seethepalli, A. , K. Dhakal , M. Griffiths , H. Guo , G. T. Freschet , and L. M. York . 2021. “Rhizovision Explorer: Open‐Source Software for Root Image Analysis and Measurement Standardization.” AoB Plants 13: plab056. 34804466 10.1093/aobpla/plab056 PMC8598384

[pce15369-bib-0067] Shangguan, W. , T. Hengl , J. Mendes de Jesus , H. Yuan , and Y. Dai . 2017. “Mapping the Global Depth to Bedrock for Land Surface Modeling.” Journal of Advances in Modeling Earth Systems 9: 65–88.

[pce15369-bib-0068] Tataranni, G. , M. Santarcangelo , A. Sofo , C. Xiloyannis , S. D. Tyerman , and B. Dichio . 2015. “Correlations between Morpho‐Anatomical Changes and Radial Hydraulic Conductivity in Roots of Olive Trees Under Water Deficit and Rewatering.” Tree Physiology 35: 1356–1365. 26446266 10.1093/treephys/tpv074

[pce15369-bib-0069] Thermo Fisher Scientific . (2019) Avizo Software for Materials Research: Materials Characterization and Quality Control (Reprint). Available online: https://assets.thermofisher.com/TFS-Assets/MSD/brochures/brochure-avizo-software-materials-research.pdf.

[pce15369-bib-0070] De Toledo, J. J. , W. E. Magnusson , C. V. Castilho , and H. E. M. Nascimento . 2011. “How Much Variation in Tree Mortality Is Predicted by Soil and Topography in Central Amazonia?” Forest Ecology and Management 262: 331–338.

[pce15369-bib-0071] Tomasella, M. , V. Casolo , N. Aichner , et al. 2019. “Non‐Structural Carbohydrate and Hydraulic Dynamics during Drought and Recovery in *Fraxinus Ornus* and *Ostrya Carpinifolia* Saplings.” Plant Physiology and Biochemistry 145: 1–9. 31665662 10.1016/j.plaphy.2019.10.024

[pce15369-bib-0072] Tomasella, M. , E. De Nardi , F. Petruzzellis , S. Andri , M. Castello , and A. Nardini . 2022. “Green Roof Irrigation Management Based on Substrate Water Potential Assures Water Saving Without Affecting Plant Physiological Performance.” Ecohydrology 15: e2428.

[pce15369-bib-0073] Tomasella, M. , E. Petrussa , F. Petruzzellis , A. Nardini , and V. Casolo . 2020. “The Possible Role of Non‐Structural Carbohydrates in the Regulation of Tree Hydraulics.” International Journal of Molecular Sciences 21: 144. 10.3390/ijms21010144 PMC6981889 31878253

[pce15369-bib-0074] Tyree, M. T. , S. Patiño , J. Bennink , and J. Alexander . 1995. “Dynamic Measurements of Roots Hydraulic Conductance Using a High‐Pressure Flowmeter in the Laboratory and Field.” Journal of Experimental Botany 46: 83–94.

[pce15369-bib-0075] Zhang, X. , S. Ma , H. Hu , F. Li , W. Bao , and L. Huang . 2024. “A Trade‐Off between Leaf Hydraulic Efficiency and Safety Across Three Xerophytic Species in Response to Increased Rock Fragment Content.” Tree Physiology 44: tpae010. 38245807 10.1093/treephys/tpae010 PMC10918055

[pce15369-bib-0076] Zwieniecki, M. A. , and M. Newton . 1995. “Roots Growing in Rock Fissures: Their Morphological Adaptation.” Plant and Soil 172: 181–187.

