# Supplementary material for: Hydraulic Properties of a Rock‐Soil‐Root System: Insights From Fraxinus ornus L. Saplings Growing on Different Carbonate Rocks
Source: Plant Cell Environ. 2025 Jan 8;48(5):3448–58. doi: 10.1111/pce.15369 (PMC11963479; doi:10.1111/pce.15369)
Supplement: Supplementary file 1 — Supporting information. [file PCE-48-3448-s001.docx]

**Supplementary Material**

**
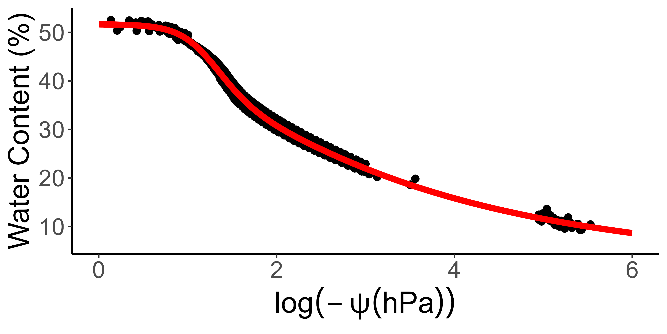
**
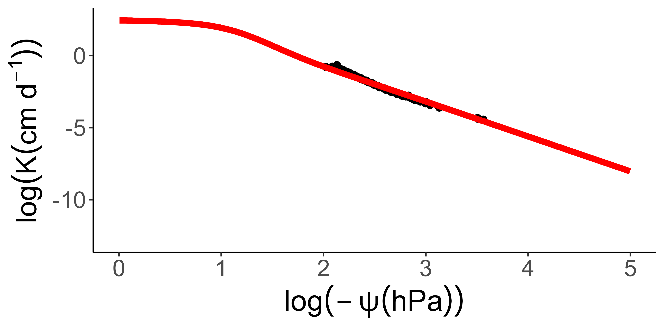


a b

Figure S1: Soil Water retention curve (a) and Soil hydraulic conductivity (b) measured with the combination of the HYPROP method together with the WP4C method. Black points are the measured data, while the red line is the corresponding fit.


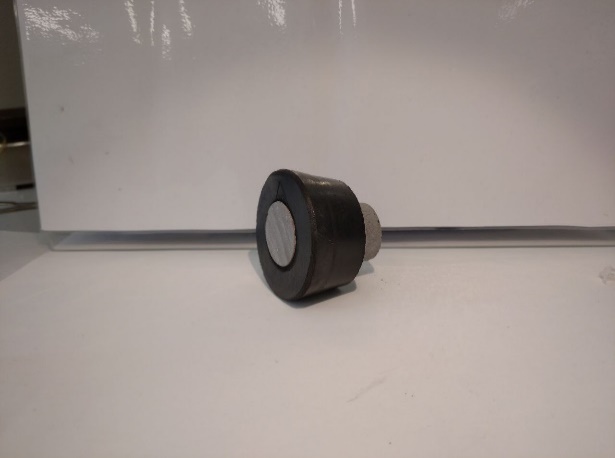

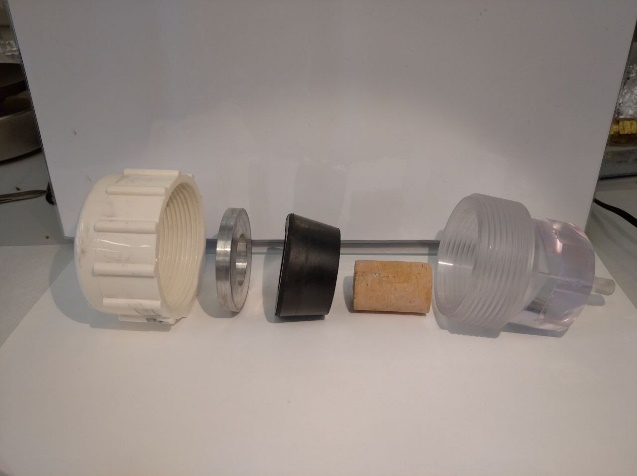


a b


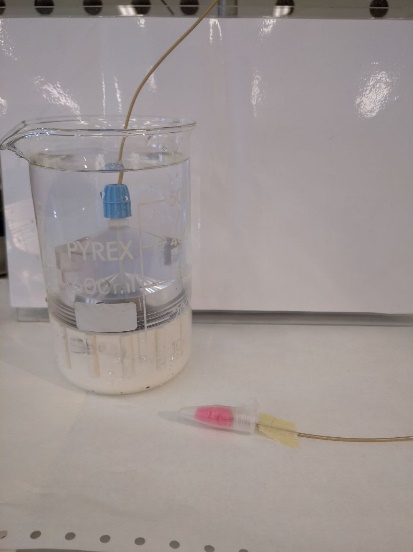

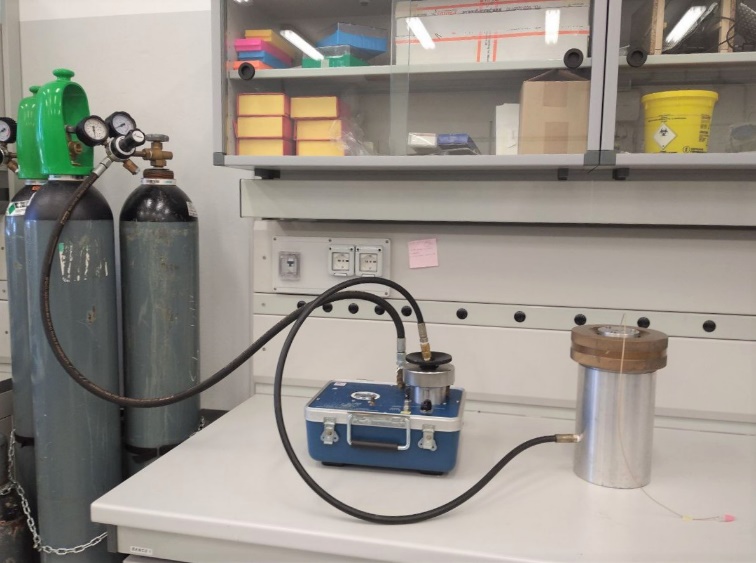


c d

Figure S2: Setup for rock hydraulic conductivity measurements: rock cylinder inserted in the gasket (a), hydraulic reducer components (b), assembled components immersed in the beaker (c), pressure chamber from which the capillary comes out and connection to the nitrogen tank (d).

|  |  |  |  |  |  |  |
| --- | --- | --- | --- | --- | --- | --- |
|  | Well Watered | | | Stressed | | |
|  | S | B | D | S | B | D |
| EL (%) | 24.2±9.8 | 18.8±7.72 | 16.4±3.3 | 5.5±1.5 | 6.0±1.1 | 5.1±0.9 |
|  |  |  |  |  |  |  |
|  |  |  |  |  |  |  |
|  |  |  |  |  |  |  |

Tab S1: Relative electrolyte leakage of root samples from well watered and stressed plants grown in pure soil (S) or soil mixed with Breccia (B) or Dolostone (D) rock fragments.

Grid Convergence Study

To validate that our results are independent of grid refinement, we conducted a grid convergence study utilizing the Grid Convergence Index (GCI) method as outlined by Roache (1994). The GCI, which is based on Richardson extrapolation (Richardson & Gaunt, 1927; Richardson & Glazebrook, 1911), quantifies the error introduced by grid resolution in numerical simulations. For this study, we created three different mesh resolutions: coarse (50x50 µm), medium (100x100 µm), and fine (200x200 µm), and ran our model on each of these meshes.

The refinement of the meshes was achieved by varying the refinement levels in snappyHexMesh, progressing from the coarse to the fine grid. Ideally, this approach would result in a grid refinement of 100x100 µm. However, due to the complexity of the image-based grids, mesh quality issues can arise during the meshing process. To meet the predefined mesh quality standards, snappyHexMesh can automatically correct these issues by refining cells or merging faces, generating a valid mesh through an iterative process. As a result, the actual grid refinement ratio differed from the theoretical value of 100x100 µm.

Therefore, we computed the effective grid refinement ratio ($r_{eff}$​) using the formula provided by Roache (1994):

$$r_{eff}= \left( \frac{N_{1}}{N_{2}} \right)^{1/D}$$

where $N_{1}$ is the number of cells in the fine grid, $N_{2}$ is the number in the medium grid, and $D$ is the dimensionality of the problem.

Next, following the approach of Roache (1997), we determined the order of convergence ():

$$p=ln\left( \frac{f_{3}-f_{2}}{f_{2}-f_{1}} \right)\cdot ln\left( r_{eff} \right)^{-1}$$

Where $f_{1}$, $f_{2}$, $f_{3}$ are the numerical solution of the integral of the water potential ($\hat{\psi} )$ in the fine, medium and coarse grid.

Since Richardson extrapolation assumes monotonic convergence of truncation errors (Roache, 1997), we evaluated the convergence ratio 𝑅 in our study as follows:

$$R= \frac{f_{2}-f_{1}}{f_{3}-f_{2}}$$

A convergence ratio of $R\approx0.18$confirmed the monotonic convergence of our problem. Subsequently, we carried out Richardson extrapolation to obtain a higher-order estimate for the parameter of interest as the grid spacing approaches zero ($h\to0$). Since our simulations were conducted using the medium grid, we applied Richardson extrapolation as described by Roache (1994) in the following manner:

$$f_{h=0}\cong f_{2}+ \frac{(f_{1}-f_{2})\cdot r_{eff}^{p}}{r_{eff}^{p}-1}$$

Next, we computed the GCI for both the fine and medium grids, considering that our study is centered on the medium grid (Roache, 1994). This approach results in a more conservative error estimate.

$${GCI}_{12}=\frac{F_{s}\cdot\left| \epsilon_{1} \right|\cdot r_{eff}^{p}}{r^{p}-1}$$

With

$$\epsilon_{1}=\frac{f_{2}-f_{1}}{f_{1}}$$

And $F_{s}=1.25$ as a safety factor. The $GCI$ for the medium and coarse grids (${GCI}_{23}$) was calculated accordingly using:

$$\epsilon_{2}=\frac{f_{3}-f_{2}}{f_{2}}$$

By comparing the two $GCI$ values derived from the three grids, we can determine if the grids fall within the asymptotic range of convergence. This condition is met if the following equation holds true:

$${CGI}_{23}{=r}_{eff}^{p}\cdot{CGI}_{12}$$

Or

$$\frac{{CGI}_{23}}{r_{eff}^{p}\cdot{CGI}_{12}}\cong1$$

The results of the grid convergence study are summarized in the Table S2:

Table S2: Results of the grid convergence study carried out for a representative sample.

|  | $\boldsymbol{r}_{\boldsymbol{eff}}\boldsymbol{[-}\boldsymbol{]}$ | $\boldsymbol{p}\boldsymbol{[-]}$ | $\boldsymbol{R}\boldsymbol{[-]}$ | $\boldsymbol{f}_{\boldsymbol{h}\boldsymbol{=0}}\boldsymbol{[}\boldsymbol{hPa}\boldsymbol{]}$ | $\boldsymbol{CGI}_{\boldsymbol{12}}\boldsymbol{[\%]}$ | $\boldsymbol{CGI}_{\boldsymbol{23}}\boldsymbol{[\%]}$ | $\frac{\boldsymbol{CGI}_{\boldsymbol{23}}}{\boldsymbol{r}_{\boldsymbol{eff}}^{\boldsymbol{p}}\boldsymbol{\cdot}\boldsymbol{CGI}_{\boldsymbol{12}}}$ |
| --- | --- | --- | --- | --- | --- | --- | --- |
| $\hat{\boldsymbol{\psi}}$ | 1.41 | 5.42 | 0.18 | $-1.25\cdot{10}^{-3}$ | 0.62 | 4.07 | 1.004 |

The convergence ratio 𝑅 of approximately 0.18 verifies the monotonic convergence of the studied problem. The GCI values indicate that the simulations are within the asymptotic range of convergence, with an error band for the volumetric flow rate calculations of approximately 0.62% for the fine-to-medium grids and 4.07% for the medium-to-coarse grids.

The results of the spatial grid convergence study suggest that our numerical simulations are independent of the mesh resolution. The error estimates are within acceptable limits, indicating reliable and accurate numerical results.

**References**

Richardson LF, Glazebrook RT. 1911. The approximate arithmetical solution by finite differences of physical problems involving differential equations, with an application to the stresses in a masonry dam. Philos Trans R Soc Lond A. 210(459-470):307-357

Richardson LF, Gaunt JA. 1927. The deferred approach to the limit. Philos Trans R Soc Lond A. 226(636-646):299-361.

Roache PJ. 1994. Perspective: A method for uniform reporting of grid refinement studies. J Fluids Eng. 116(3):405-413

Roache PJ. 1997. Quantification of uncertainty in computational fluid dynamics. Annu Rev Fluid Mech. 29(1):123-160
